# Non-destructive elemental analysis of a carbonaceous chondrite with direct current Muon beam at MuSIC

**DOI:** 10.1038/s41598-017-15719-5

**Published:** 2017-11-13

**Authors:** K. Terada, A. Sato, K. Ninomiya, Y. Kawashima, K. Shimomura, G. Yoshida, Y. Kawai, T. Osawa, S. Tachibana

**Affiliations:** 1 Graduate School of Science, Osaka University, Osaka, Japan; 2 High Energy Accelerator Research Organization, Tsukuba, Japan; 3 Material Sciences Research Center, Japan Atomic Energy Agency, Ibaraki, Japan; 4 Graduate School of Science, Hokkaido University, Sapporo, Japan; 5 Graduate School of Science, The University of Tokyo, Tokyo, Japan

## Abstract

Electron- or X-ray-induced characteristic X-ray analysis has been widely used to determine chemical compositions of materials in vast research fields. In recent years, analysis of characteristic X-rays from muonic atoms, in which a muon is captured, has attracted attention because both a muon beam and a muon-induced characteristic X-ray have high transmission abilities. Here we report the first non-destructive elemental analysis of a carbonaceous chondrite using one of the world-leading intense direct current muon beam source (MuSIC; MUon Science Innovative Channel). We successfully detected characteristic muonic X-rays of Mg, Si, Fe, O, S and C from Jbilet Winselwan CM chondrite, of which carbon content is about 2 wt%, and the obtained elemental abundance pattern was consistent with that of CM chondrites. Because of its high sensitivity to carbon, non-destructive elemental analysis with a muon beam can be a novel powerful tool to characterize future retuned samples from carbonaceous asteroids.

## Introduction

The muon is one of the charged leptons, and has a mass of 105.7 MeV/*c* ^2^, approximately 200 times heavier than the electron. Because muons have much higher transmissivity than electrons and X-rays, cosmic-ray muon tomography/radiography has been used to image the interiors of volcanoes^1^, nuclear waste^2^ and Fukushima nuclear reactors^3^.

Not only cosmic-ray induced natural muons but an artificial muon beam produced in acceleration facilities also has a huge potential to probe the interior of materials without destruction and severe radiation damage as proposed by Rosen *et al*.^4^.

Muons can penetrate much deeper into materials than electrons because of less effective bremsstrahlung process due to their heavier mass. On the other hand, they lose their momentum eventually and stop at certain depth depending on the density of target material and the initial momentum. In the classical Bohr model, orbital radii of negative leptons (electron and/or muon) around an atomic nucleus are inversely proportional to the lepton mass, and therefore a muon trapped by an atom has an orbit closer to the atomic nucleus than electrons due to its ~200 times heavier mass. A muon, first trapped in the outer loosely bound orbit, makes a cascade transition to the muonic 1 s orbit, resulting in the emission of characteristic muonic X-rays. Because the energy gaps between orbits are proportional to the lepton mass, the characteristic muonic X-ray has an energy ~200 times larger than that associated with the orbital transition of electrons. For instance, electron-induced Kα–X-rays of C, O, Si, and Fe that are used for energy-dispersive X-ray spectroscopy (EDS) on scanning electron microscopes (SEM) and/or electron microprobes (EPMA) have energies of 0.3, 0.5, 1.7, and 6.4 keV, respectively, whereas muonic Kα–X-rays of C, O, Si, and Fe have energies of 75, 134, 400, and 1256 keV, respectively. Such high-energy characteristic muonic X-rays can pass through the 1cm-size material without significant absorption. These features of muon and muon-induced characteristic X-rays have attracted an interest in application to non-destructive elemental analysis of novel samples.

In November 2009, J-PARC MUSE (Japan Proton Accelerator Research Complex, MUon Science Establishment) succeeded in providing a decay muon rate of 10^6^ muons/s for a momentum of 60 MeV/*c*, which has been the most intense “pulsed” muon beam in the world (25 Hz packet of muon cloud^5^). This intense pulsed muon beam has been used for various novel applications, one of which is non-destructive chemical analysis^6–9^. Terada *et al*.^7^ succeeded in detection of muon-induced characteristic X-rays of light elements (B, C, N, and O) and their depth distributions from a several mm-thick layered sample. They also made non-destructive bulk elemental analysis of meteorites containing organic materials, and demonstrated the potential of application to non-destructive analysis of future asteroidal returned samples. Osawa *et al*.^8^ reported further technical development for the elemental analysis at J-PARC MUSE.

Recently, a “direct-current” muon source, MuSIC (MUon Science Innovative Channel), was newly established at RCNP (Research Centre for Nuclear Physics), Osaka University to generate the most intense direct-current (DC) muon beam with the extremely high proton-to-muon yield (10^8^ muons/s with a 0.4 kW proton beam^10^). Since 2013 MuSIC has provided an intense DC muon beam with a momentum range of 20–120 MeV/*c* ^11,12^, which can also be applicable for non-destructive elemental analysis. Here we report a result of muon-induced characteristic X-ray spectroscopy of Jbilet Winselwan meteorite with the MuSIC DC muon beam. Jbilet Winselwan meteorite is classified into the CM group of carbonaceous chondrites, and contains ~2 wt% of carbon dominantly as organic matter^13^. The carbon concentration of 2 wt% is typical for CM chondrites. CM chondrites can be a good analogue of samples that are expected to be returned from C-type and B-type asteroids in 2020’s^14,15^, and this study could be a good test to study the potential of the elemental analysis with a direct-current muon beam for the analysis of future retuned samples.

## Results

### Non-destructive bulk elemental analysis of carbonaceous chondrite

**Figure 1:**
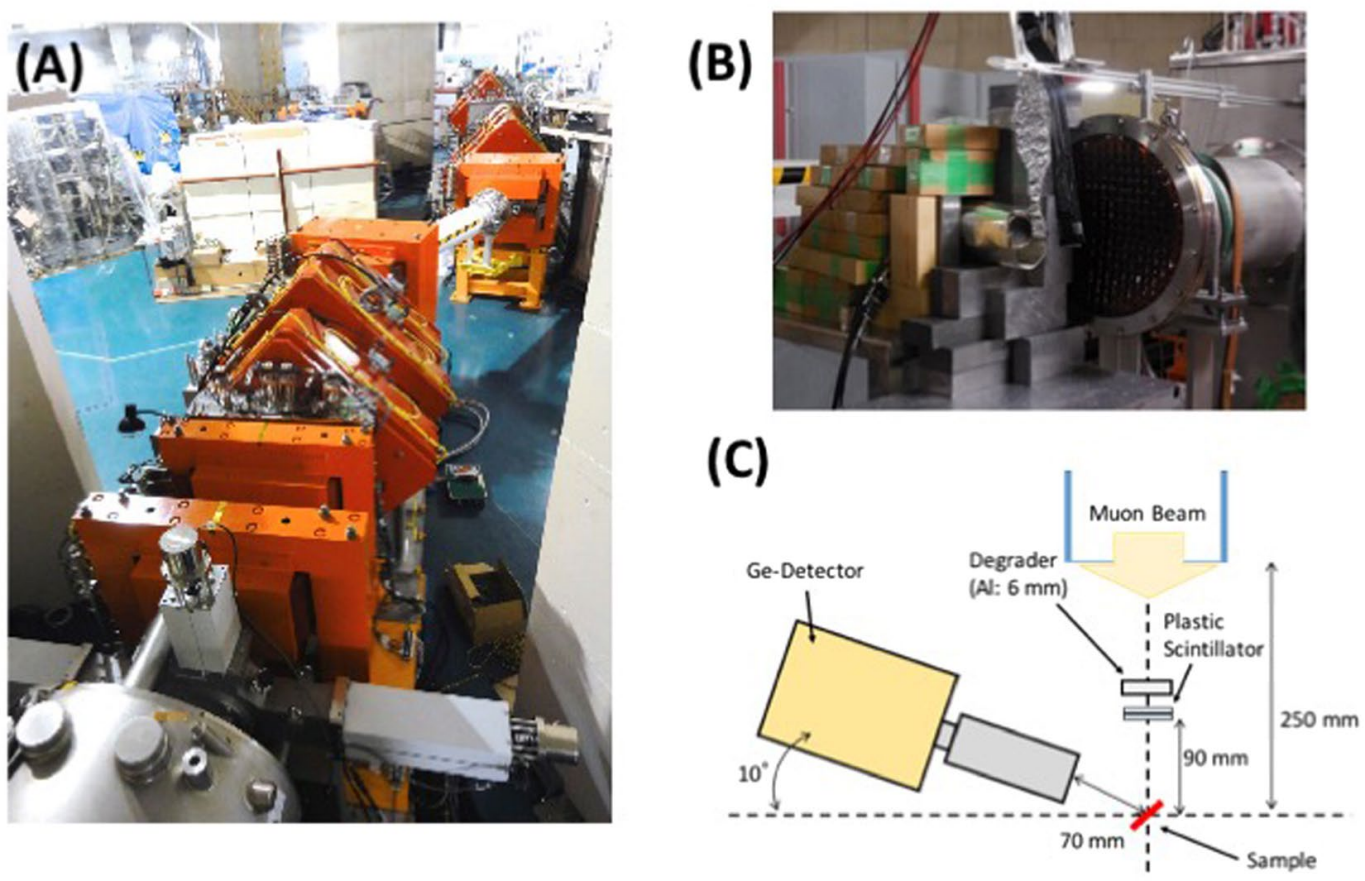
(**A**) An entire view of the MuSIC (MUon Science Innovative Channel) beam line at RCNP (Research Centre for Nuclear Physics), Osaka University. (**B**) The outlet of muon beam at the MuSIC. (**C**) A schematic illustration of analytical setting.

**Figure 2:**
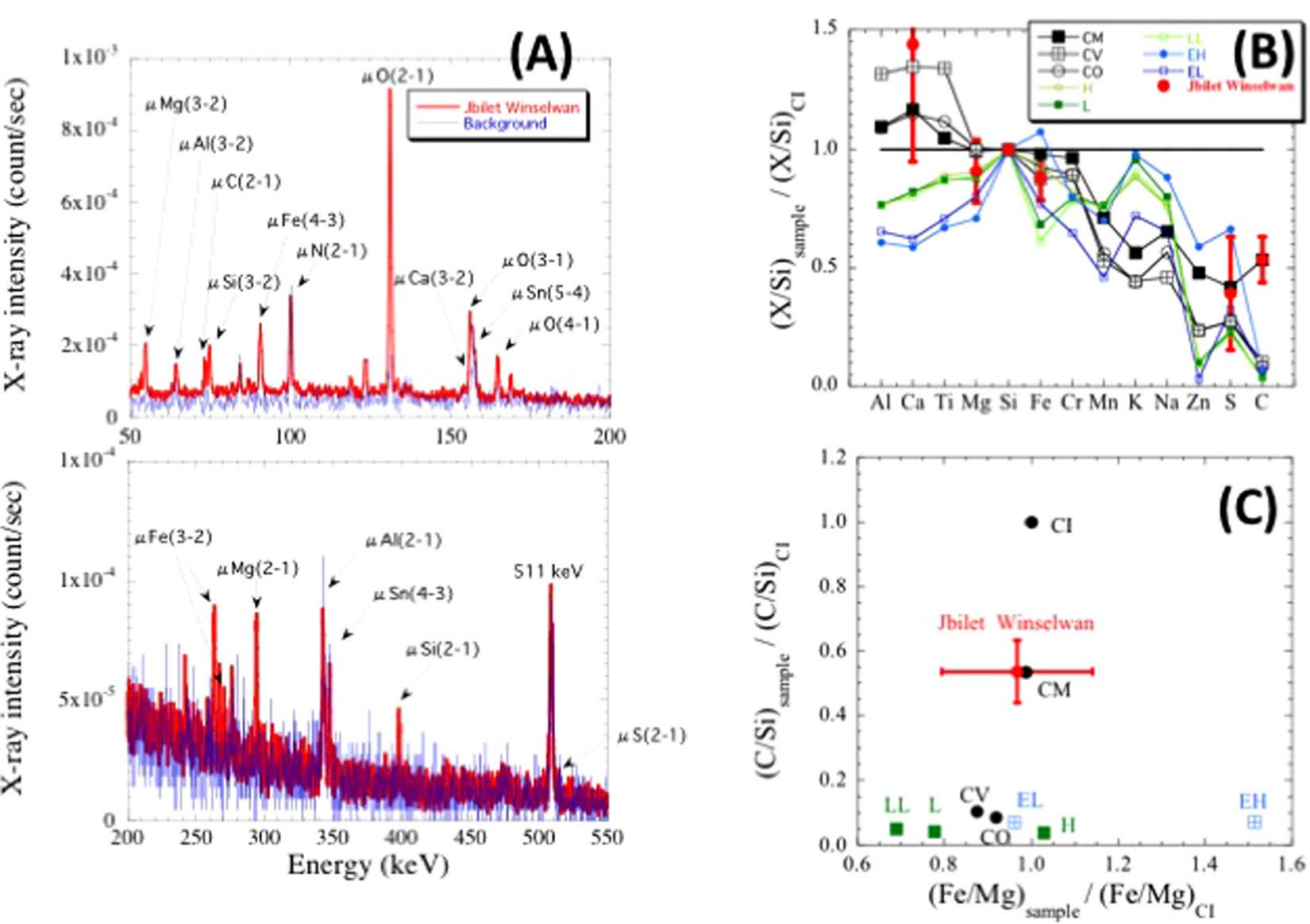
(**A**) Muonic X-ray energy spectra from Jbilet Winselwan (CM2) and for background measurement. (**B**) Comparison of element/Si ratios of Jbilet Winselwan, normalized to those of CI chondrites, with the elemental abundance patterns of different chemical groups of chondrites^16^. (**C**) Comparison of CI-normalized C/Si and Fe/Mg ratios of Jbilet Winselwan with those of different chemical groups of chondrites^16^.

A 3 cm × 3 cm × 0.6 cm chip of Jbilet Winselwan meteorite was prepared for the bulk elemental analysis at the MuSIC facility (Fig. 1). The meteorite chip was exposed to the muon beam with the momentum of 60 MeV/*c* for about 20 hours, and a muonic X-ray spectrum emitting out from the ~3-mm depth of the sample was obtained with a high-purity germanium detector. A comparison of the muonic X-ray spectrum from Jbilet Winselwan with the background spectrum (Fig. 2A) shows clear detection of muonic X-rays of Mg, C, Si, O and Fe and marginal detection of those of Ca and S from the meteorite sample. We especially note that the C-K_α_ signal at 75 keV from 2 wt% of carbon in the sample is clearly distinct from that of Si-L_α_ at 77 keV. We also note that the 511 keV peak is that for positron annihilation and that peaks for Al, Sn, and N were mainly from a sample holder, a masking shield, and the atmosphere, respectively. The background-subtracted net signals of muonic characteristic X-rays from Jbilet Winselwan are listed in Table 1.

**Table-1:** Characteristic muonic X-ray counts of Jbilet Winselwan and the normalized intensity by Si(3-2).

Characteristic X-ray	Energy (keV)	Intensity (c/s)	Normalized Intensity by Si (3-2)
Mg(3-2)	56.6	0.279 ± 0.022	0.896 ± 0.092
C(2-1)	75.2	0.264 ± 0.026	0.849 ± 0.100
Si(3-2)	76.7	0.311 ± 0.021	
Fe(4-3)	92.6	0.464 ± 0.027	1.489 ± 0.133
O(2-1)	133.2	2.106 ± 0.077	6.764 ± 0.516
Ca(3-2)	156.0	0.133 ± 0.028	0.428 ± 0.095
Fe(3-2)	265.3	0.904 ± 0.076	2.904 ± 0.311
Mg(2-1)	296.3	0.667 ± 0.067	2.143 ± 0.258
Si(2-1)	400.3	0.560 ± 0.080	1.797 ± 0.284
S(2-1)	515.6	0.166 ± 0.076	0.532 ± 0.246

Because the muonic X-rays from the 3-mm depth of the sample can pass through the sample without significant absorption^7^, the obtained muonic X-ray intensities should represent the elemental abundances of Jbilet Winselwan. In order to obtain the elemental abundance ratios of Jbilet Winselwan from the observed muonic X-ray intensities, we first estimated the sensitivity factor of muonic X-ray intensity of each element relative to that of Si(3-2) using the intensity data obtained at J-PARC MUSE from a Murchison CM2 chondrite^7^. The sensitivity factor is given by ${f}_{X/Si(3\mbox{--}2)}=({A}_{X}/{A}_{Si})/({I}_{X(m\mbox{--}n)}/{I}_{Si(3\mbox{--}2)})$, where ${A}_{X}/{A}_{{Si}}$ is the atomic abundance ratio of an element *X* relative to Si in Murchison^16^ and ${I}_{X(m\mbox{--}n)}/{I}_{Si(3\mbox{--}2)}$ represents the intensity ratio of muonic X-ray for the transition of muon from the principal number *m* to *n* in the element *X* relative to that of Si(3-2).

Assuming that the sensitivity factors for Murchison meteorite at J-PARC MUSE are applicable to this study, the elemental abundance ratios of Jbilet Winselwan were calculated and are shown with those of different chemical groups of chondrites^16^ in Fig. 2B and C. Because two characteristic X-rays from muonic Mg were detected (Mg(2-1) and Mg(3-2); Table 1), the average of two ${A}_{{Mg}}/{A}_{{Si}}$ ratios, which were independently calculated from two transitions, was adopted to obtain the Mg/Si ratio of the meteorite. Because of low count rates of muonic X-rays under the present analytical condition, analytical uncertainties shown in Fig. 2B and C were obtained from propagation of counting statistical uncertainties of muonic characteristic X-rays for the analysis of both Jbilet Winselwan in this study and Murchison in J-PARC^7^. The analysis with multiple detectors covering a wide solid angle will improve the analytical uncertainties in future studies.

Although the sensitivity factors should be determined under the analytical conditions with MuSIC for detailed comparison, the estimated abundance pattern of Jbilet Winselwan (especially C/Si ratio) well matches with that of CM chondrites^16^, consistent with the chemical classification in previous studies^13,17^.

### Muon irradiation to organic compounds

**Figure 3:**
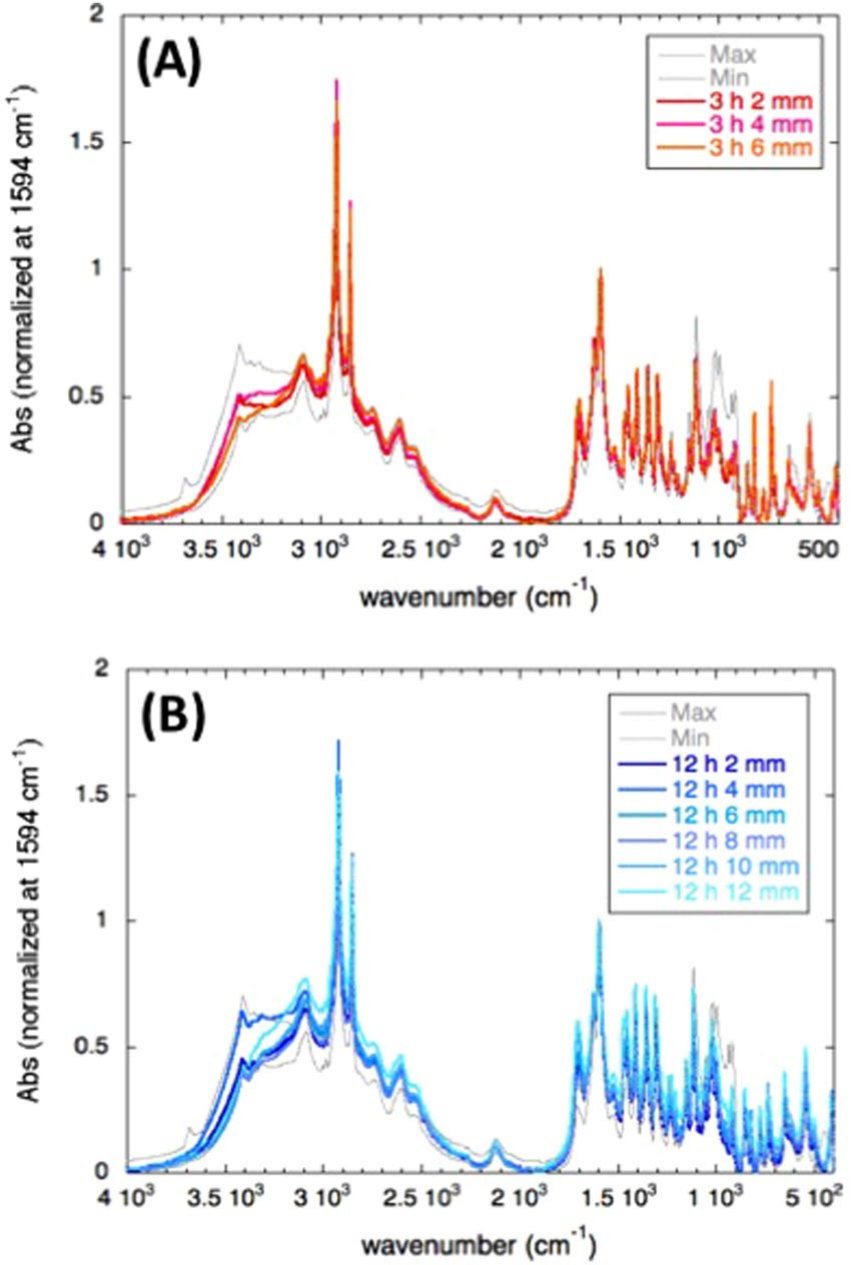
Infrared spectra of a mixture of organic compounds (alanine, glucose, paraformaldehyde, phenanthrene, and stearic acid), exposed for different durations ((**A**) 3 hours and (**B**) 12 hours) and at different depths of the sample pellet. The maximum and minimum absorbance obtained from multiple analysis of the starting mixture (non-exposed samples) are also shown for comparison.

Carbonaceous chondrites contain carbon dominantly as organic compounds, and future asteroidal returned samples are also expected to contain organic matter^14,15^. Although the muon induced elemental analysis does not cause significant irradiation damage to the samples, pellets of mixed organic chemical reagents (alanine, glucose, paraformaldehyde, phenanthrene, and stearic acid) were exposed to the muon beam for 3–12 hours to check the irradiation damage. Infrared spectra of exposed pellets are shown in Fig. 3 with the maximum and minimum absorbance obtained from non-exposed starting mixtures of reagents. The comparison between the exposed pellets and non-exposed starting materials suggests that infrared spectra of the irradiated samples for different durations and at different depths from the surface do not show any systematic changes with either the exposure time or the depth, and are not different from those of non-exposed samples within the variation of initial reagent mixtures. Although more rigorous investigation is needed to examine the damage to individual molecules if one is interested in specific compounds, we conclude that the muon irradiation has little or no severe effects on most of bond structures in organic compounds under these analytical conditions compared to synchrotron X-ray irradiation^18,19^.

## Discussion

The elements with concentrations higher than 1 wt% were detected in this experiment. This is consistent with the previous work^7^, where Murchison and Allende carbonaceous chondrites were analyzed at J-PARC MUSE. The detection limit in the present study is mainly attributed to the X-ray detection setting with a single Ge detector. In this study, the incident muon flux through the plastic scintillators was about 250 counts/s, of which cross section is 5 cm × 5 cm, while the gross X-ray counts from the sample detected coincidently with scintillator signals were ~2.3 counts/s, of which cross section was 2 cm × 3 cm (about one-forth of that of plastic scintillator). This ~4-% detection efficiency, which is obtained from the count ratio between the muonic X-rays and the plastic scintillator (2.3 cps/250 cps) divided by the ratio of their cross sections (~1/4), is roughly comparable to the solid angle of ~3% covered with the Ge detector. Therefore, a multiple-detector system that covers a much larger solid angle is expected to easily improve the sensitivity and/or detection limit. Furthermore, the MuSIC DC muon beam intensity has recently become 50-times more intense than our analysis in 2015 with planned technical developments. This will also make a huge contribution to lower the detection limit and increase the sensitivity.

This study demonstrated that non-destructive elemental analysis of a carbonaceous chondrite with the MuSIC muon beam can detect muonic X-rays from carbon at the 3-mm depth of the sample and that the semi-quantitatively estimated carbon abundance is consistent with that previously reported^13^. The on-going asteroidal sample return missions (Hayabusa2 and OSIRIS-REx) will collect millimeter- to centimeter-sized samples at near-Earth C-type and B-type asteroids Ryugu and Bennu^14,15,20,21^ and will return the samples in 2020 and 2023, respectively. The present study proved that the bulk elemental analysis with a muon beam could be a powerful non-destructive analytical technique to compare the returned samples with known chemical groups of chondrites and to determine the carbon content in the samples.

## Methods

The MuSIC facility at Research Center for Nuclear Physics (RCNP), Osaka University, Japan, was designed to produce the direct current muon beam with the world-highest proton to muon yield^22^. A continuous proton beam is supplied to a cylindrical graphite target (4 cm in diameter and 20 cm in length) by a cyclotron to produce pions that decay into muons and muon neutrinos. The intensity and energy of the proton beam are 1.1 μA and 392 MeV, respectively, and the beam power is 0.431 kW that is much smaller than those at other synchrotron facilities^10^. In order to produce an intense muon beam from the ~0.4 kW proton beam, a novel pion capture system is employed, and the MuSIC can produce a direct current muon beam with the intensity of ~10^5^ muons/s with the momentum range of 24–110 MeV/*c* ^10^. The momentum peak of the MuSIC muon beam at the current setting is ~70 MeV/*c* with a beam size of 5 cm with a FWHM.

The analysis of a chip of Jbilet Winselwan (3 cm × 3 cm × 0.6 cm) was conducted at RCNP in November 2015 (E411: Development on non-destructive elemental analysis of planetary materials by using high intensity μ^−^ beam, PI: K. Terada). The sample was placed using a holder made of aluminum foil at the distance of 250 mm from the outlet of muon beam. A high-purity germanium detector (CANBERRA, BE2020) with a diameter of 51.5 mm was used for the X-ray detection. The detector was placed at the distance of 70 mm from the sample. The sample and the Ge detector were oriented at 45 and 80 degrees to the muon beam, respectively (Fig. 1C).

The primary proton beam intensity was 20 nA, and its intensity fluctuation was monitored using a secondary electron counter located near the pion production target. The muon momentum was set at 60 MeV/*c* to obtain an appropriate muon beam intensity. The muon momentum of 60 MeV/*c* corresponds to the stopping depth of about 9.4 mm in CM chondrites (density ~2.1 g/cm^3^), which is larger than the sample thickness (6 mm). In order to reduce the stopping distance down to ~3 mm from the sample surface, a 6-mm thick aluminum plate was placed in front of the sample as a degrader (Fig. 1C). A pair of plastic scintillators that detect the muon passage was also placed in front of the sample to trigger the X-ray counting system. By counting X-ray signals coincident with the signal from the plastic scintillators, we could reduce the signal-to-noise ratio dramatically. The meteorite chip was exposed to the muon beam twice (9.5 and 10.4 hours; the total exposure time of ~20 hours). The background X-ray spectrum was also taken for 5 hours in the same analytical session.

We also irradiated the muon beam to a mixture of organic compounds in order to investigate the irradiation damage. Reagent powder of alanine, glucose, paraformaldehyde, phenanthrene, and stearic acid were mixed with a weight ratio of 1:1:1:1:1 and were pressed into pellets (10 mm in diameter and ~2 mm in thickness). Three or six pellets were piled up along the beam direction and were exposed to the muon beam for 3, 6, 9 and 12 hours. Infrared spectra of exposed and non-exposed pellets were obtained with a KBr method using a FTIR spectrometer (JASCO FT/IR-4200).
